# Correction: Mindbench.ai: an actionable platform to evaluate the profile and performance of large language models in a mental healthcare context

**DOI:** 10.1038/s44277-025-00054-9

**Published:** 2026-02-02

**Authors:** Bridget Dwyer, Matthew Flathers, Akane Sano, Allison Dempsey, Andrea Cipriani, Asim H. Gazi, Bryce Hill, Carla Gorban, Carolyn I. Rodriguez, Charles Stromeyer, Darlene King, Eden Rozenblit, Gillian Strudwick, Jake Linardon, Jiaee Cheong, Joseph Firth, Julian Herpertz, Julian Schwarz, Khai Truong, Margaret Emerson, Martin P. Paulus, Michelle Patriquin, Yining Hua, Soumya Choudhary, Steven Siddals, Laura Ospina Pinillos, Jason Bantjes, Stephen M. Schueller, Xuhai Xu, Ken Duckworth, Daniel H. Gillison, Michael Wood, John Torous

**Affiliations:** 1 Division of Digital Psychiatry, Beth Israel Deaconess Medical Center, Harvard Medical School, Boston, MA USA; 2 Department of Electrical and Computer Engineering, Rice University, Houston, TX USA; 3 Department of Psychiatry and Family Medicine, School of Medicine, Centers for American Indian and Alaska Native Health, Colorado School of Public Health, Telemedicine Helen and Arthur E. Johnson Depression Center, University of Colorado Anschutz Medical Campus, Aurora, CO USA; 4 Department of Psychiatry, University of Oxford, UK; Oxford Precision Psychiatry Lab, National Institute for Health and Care Research (NIHR) Oxford Health Biomedical Research Centre, Oxford, UK; 5 School of Engineering and Applied Sciences, Harvard University, Cambridge, MA USA; 6 The Brain and Mind Centre, The University of Sydney, Camperdown, New South Wales, Australia; 7 Department of Psychiatry and Behavioral Sciences, Stanford University, Stanford, CA USA; 8 Peer Advisory Advocacy and Research Council, Massachusetts Mental Health Center Public Psychiatry Division of the Beth Israel Deaconess Medical Center, Boston, MA USA; 9 Department of Psychiatry, The University of Texas Southwestern Medical Center, Dallas, TX USA; 10 Centre for Addiction and Mental Health, Toronto, Ontario, Canada; 11 School of Psychology, Deakin University, Geelong, VIC Australia; 12 Division of Psychology and Mental Health, University of Manchester, Manchester Academic Health Science Centre, Manchester, UK; 13 Department of Psychiatry and Neuroscience, Campus Benjamin Franklin, Charité–Universitätsmedizin Berlin, Berlin, Germany; 14 Department of Psychiatry and Psychotherapy, Center for Mental Health, Immanuel Hospital Rüdersdorf, Brandenburg Medical School Theodor Fontane, Rüdersdorf, Germany; 15 College of Nursing, University of Nebraska Medical Center, Omaha, USA; 16 Laureate Institute for Brain Research, Tulsa, OK USA; 17 Baylor College of Medicine, One Baylor Plaza, Houston, TX USA; 18 Department of Epidemiology, Harvard T.H. Chan School of Public Health, Boston, MA USA; 19 Department of Psychiatry, National Institute of Mental Health and Neurosciences, Bangalore, India; 20 Institute for Life Course Health Research, Department of Global Health, Faculty of Medicine and Health Sciences, Stellenbosch University, Stellenbosch, South Africa; 21 Department of Psychiatry and Mental Health, Faculty of Medicine, Pontificia Universidad Javeriana, Bogota, Colombia; 22 Department of Psychological Science, University of California, Irvine, CA USA; 23 Department of Biomedical Informatics, Columbia University, NY USA; 24 National Alliance on Mental Illness (NAMI), Arlington, VA USA

**Keywords:** Translational research, Population screening

Correction to: *NPP—Digital Psychiatry and Neuroscience* (2025) **3**:28; 10.1038/s44277-025-00049-6; Article published online XX Month XXXX

In this article the author’s name Stephen M. Schueller was incorrectly written as Steven Scheuller.

The original article has been corrected.

